# Cystic vestibular schwannoma – a subgroup analysis from a comparative study between radiosurgery and microsurgery

**DOI:** 10.1007/s10143-024-02495-w

**Published:** 2024-06-25

**Authors:** Sophie Shih-Yüng Wang, Ahmed Rizk, Florian H. Ebner, Albertus van Eck, Georgios Naros, Gerhard Horstmann, Marcos Tatagiba

**Affiliations:** 1 Department of Neurosurgery, Eberhard Karls University, Hoppe- Seyler-Strasse 3, Tubingen, Germany; 2 Gamma Knife Center Krefeld, Krefeld, Germany; 3 Present Address: Department of Neurosurgery, Krankenhaus der Barmherzigen Brüder Trier, Trier, Germany; 4 Present Address: Department of Neurosurgery, Alfried Krupp Hospital, Essen, Germany

**Keywords:** Vestibular schwannoma, Acoustic neuroma, Outcome, Tumor recurrence, Stereotactic radiosurgery, Microsurgery

## Abstract

Some vestibular schwannoma (VS) show cystic morphology. It is known that these cystic VS bear different risk profiles compared to solid VS in surgical treatment. Still, there has not been a direct comparative study comparing both *SRS* and *SURGERY* effectiveness in cystic VS. This retrospective bi-center cohort study aims to analyze the management of cystic VS compared to solid VS in a dual center study with both microsurgery (*SURGERY*) and stereotactic radiosurgery (*SRS*). Cystic morphology was defined as presence of any T2-hyperintense and Gadolinium-contrast-negative cyst of any size in the pre-interventional MRI. A matched subgroup analysis was carried out by determining a subgroup of matched *SURGERY-*treated solid VS and *SRS*-treated solid VS. Functional status, and post-interventional tumor volume size was then compared. From 2005 to 2011, *N* = 901 patients with primary and solitary VS were treated in both study sites. Of these, 6% showed cystic morphology. The incidence of cystic VS increased with tumor size: 1.75% in Koos I, 4.07% in Koos II, 4.84% in Koos III, and the highest incidence with 15.43% in Koos IV. Shunt-Dependency was significantly more often in cystic VS compared to solid VS (*p* = 0.024) and patients with cystic VS presented with significantly worse Charlson Comorbidity Index (CCI) compared to solid VS (*p* < 0.001). The rate of GTR was 87% in cystic VS and therefore significantly lower, compared to 96% in solid VS (*p* = 0.037). The incidence of dynamic volume change (decrease and increase) after *SRS* was significantly more common in cystic VS compared to the matched solid VS (*p* = 0.042). The incidence of tumor progression with *SRS* in cystic VS was 25%. When comparing EOR in the *SURGERY-*treated cystic to solid VS, the rate for tumor recurrence was significantly lower in GTR with 4% compared to STR with 50% (*p* = 0.042). Tumor control in cystic VS is superior in *SURGERY*, when treated with a high extent of resection grade, compared to *SRS*. Therapeutic response of *SRS* was worse in cystic compared to solid VS. However, when cystic VS was treated surgically, the rate of GTR is lower compared to the overall, and solid VS cohort. The significantly higher number of patients with relevant post-operative facial palsy in cystic VS is accredited to the increased tumor size not its sole cystic morphology. Cystic VS should be surgically treated in specialized centers.

## Introduction

Vestibular schwannoma (VS) are benign tumors that arise from the vestibulocochlear nerve complex and represent ca. 90% of all neoplasms occupying the cerebellopontine angle (CPA) [1, 2]. VS can show different morphological characteristics (e.g. size, CPA extension, intracanalicular extension, flow-voight, etc.), but the best known magnetic resonance imaging (MRI) feature in VS are the development of cysts, which are visible in T2-weighted MRI. Cystic VS are associated with either intra- or extratumoral cysts that develop in the loosely organized Antoni B tissues [3]. VS cysts are thought to arise from recurrent microbleeding or osmosis-induced expansion of cerebrospinal fluid trapped in arachnoid tissue, leading to T2 hyperintense signal and variable T1 signal [4–6]. 

Even though the exact pathophysiology of these evolved cysts are not definitely known, its direct consequence into patient management has already been described and discussed in the past: Cystic VS can demonstrate a more rapid growth and expansion [7], enhanced peritumoral adhesion [8–10], worse post-operative facial nerve outcome [11]. Concordant to solid VS, both radiosurgery (*SRS*) and microsurgical resection (*SURGERY*) are possible treatment modalities.

Several studies have shown safety and effectiveness of *SRS* in cystic VS, one even going so far to state that cystic VS respond better to *SRS* due to the more pronounced volume reduction VS compared to solid VS [4, 12, 13]. Still, there has not been a direct comparative study comparing both *SRS* and *SURGERY* effectiveness in cystic VS. This study aims to analyze the management of cystic VS compared to solid VS in a dual center study of *SURGERY* and *SRS* treated VS patients.

## Methods

### Study design & patient cohort

This is a retrospective dual-center cohort study. Patients were identified by a prospectively kept registry by both senior authors (G. H. and M. T.). Data was then retrospectively collected from both tertiary and specialized centers involved in the treatment of VS between 2005 und 2011 to enable follow-up (FU) of up to 10 years. Part of this data has been published before as this is a subgroup analysis [14]. 

### Treatment modalities

Patients treated by *SURGERY* were all operated by retrosigmoid approach using intraoperative electrophysiological monitoring. Patients were either operated in semi-sitting or supine position [15]. All VS patients in the *SRS* cohort received Gamma-Knife-Radiosurgery (GKR - Elekta AB, Stockholm, Sweden) with a prescription dose of 13 Gy to the 65% isodose line as a standard dose and a mean Paddick Conformity Index of 0.81 (± SD0.11) in the whole cohort. The treatment plan spared the cochlea in order to enable a higher chance on hearing preservation.

### Data collection

Cystic morphology was defined as presence of T2-hyperintense and Gadolinium-contrast-negative cyst of any size in the pre-interventional MRI. Tumor size was classified by Koos Classification [16]. Previously treated VS and VS associated with Neurofibromatosis were systematically excluded. Clinical state was reported by House and Brackmann (H&B) and Gardner-Robertson (G&R) scale (with H&B and G&R 1–2 considered as good functional outcome), and Recurrence-free-survival (RFS) was assessed radiographically by contrast-enhanced MR imaging [17, 18]. The criteria for tumor recurrence/progression was defined as a progredient growth in Gadolinium contrast-enhanced MRI (radiographic tumor control = RTC). To exclude the previously described phenomenon of pseudoprogression after *SRS* of VS, patients with tumor volume (TV) increase 6 months after *SRS* with stable TV afterwards or TV decrease were not graded as VS recurrence/progression [19]. The TV was measured using slice-by-slice manual contouring. In *SRS*, pseudoprogression was defined as transient tumor volume increase > 30% within the first two years after *SRS*-treatment. Early recurrence was defined as tumor volume increase > 30% persisting over 2 years after treatment.

In case of *SURGERY*, extent of resection (EOR) was classified by first post-operative Gadolinium-enhanced MRI 3 months post-operatively in the following manner: residual contrast-enhancing tumor was defined as subtotal resection (STR), whereas gross total resection (GTR) was defined as lack of contrast-enhancement in the MRI. Secondary VS symptoms like trigeminal affection, tinnitus and vertigo were also collected retrospectively.

### Statistical analysis

A matched subgroup analysis (1:1) was carried out by determining a subgroup of *SURGERY-*treated solid VS (closest match for tumor size, age, sex, Charlson Comorbidity Index (CCI) [20] and EOR) and *SRS*-treated solid VS (closest match for tumor size, age, sex, and CCI). Functional status, and post-interventional tumor volume size was then compared.

Statistical analysis was performed in R Studio (Version 1.2) using descriptive statistics. To compare non-numeric parameters of both groups, the chi-square test, and Fisher-T-test (for small sample size) was applied. For numeric parameters, Welch’s two sample t-test was used. Recurrence/Progression-free survival was estimated using the Kaplan-Meier method and compared between cases and controls using a log-rank test. The length of FU for recurrence/progression-free survival was calculated from the date of surgical intervention to the date of either recurrence/progression or the last clinical visit. Significance was defined as the probability of a two-sided type 1 error being < 5% (*p* < 0.05). Data is presented as mean ± standard deviation (SD) if not indicated otherwise.

## Results

### Study cohort

**Fig. 1:**
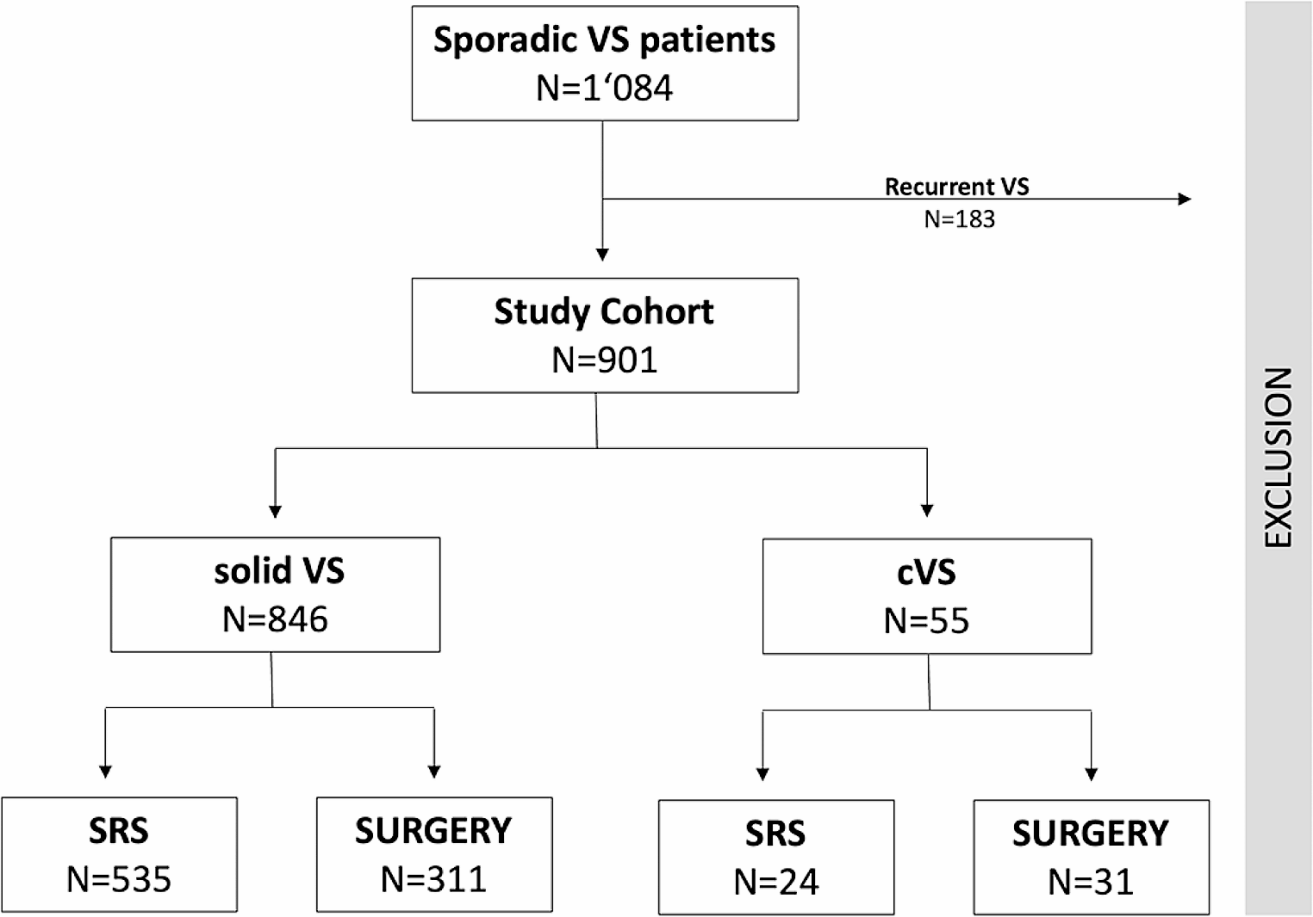
Patient cohort flowchart

From 2005 to 2011, *N* = 901 patients with primary and solitary VS were treated in both centers. 6% showed cystic morphology at the pre-interventional MRI, while 94% VS were classified as solid VS (Fig. 1). The rate of *SURGERY* was significantly higher in cystic VS with a rate of 56%, compared to 37% in solid VS (*p* = 0.006).

Mean age of patients with solid VS was 54.54 (± 13.52) years and for cystic VS 54.93 (± 16.42) years. There was no significant difference in age, when comparing the cohort of cystic with solid VS (*p* = 0.838). Cystic VS significantly more frequently reached size Koos IV (Table 1). Therefore, the incidence of cystic VS increased with tumor size: 1.75% in Koos I, 4.07% in Koos II, 4.84% in Koos III, and the highest incidence with 15.43% in Koos IV. Shunt-Dependency was significantly more prevalent in cystic compared to solid VS (*p* = 0.024) and patients with cystic VS presented with significantly worse CCI compared to solid VS (*p* < 0.001). The incidence of recurrence/progression was higher in cystic VS (16%) compared to solid VS (9%), but this did not reach statistical significance. The incidence of pre-operative trigeminal neuralgia and vertigo as secondary VS-related symptoms were significantly higher in cystic VS compared to solid VS.

**Table 1:** Tumor and patient demographics solid VS vs. cVS

	All (*N* = 901)	solid VS (*N* = 846)	cVS (*N* = 55)	*p*-value
Age	54.47(± 13.70)	54.54(± 13.52)	54.93(± 16.42)	0.838
Female	502 (56)	467 (55)	35 (64)	0.262
Koos Classification				
Koos I	114 (12)	112 (13)	2 (4)	**0.035**
Koos II	295 (33)	283 (34)	12 (22)	0.077
Koos III	330 (37)	314 (37)	16 (29)	0.251
Koos IV	162 (18)	137 (16)	25 (45)	**< 0.001**
Shunt-Dependency	19 (2)	15 (2)	4 (7)	**0.024**
Recurrence / Progression	84 (9)	75 (9)	9 (16)	0.088
Comodbidities				
Charlson Index	0.22(± 0.73)	0.20(± 0.68)	0.55(± 1.20)	**< 0.001**
0	807 (90)	764 (90)	43 (79)	**0.009**
1	33 (4)	30 (4)	3 (5)	0.447
2	35 (4)	31 (4)	4 (7)	0.159
3	11 (1)	9 (1)	2 (4)	0.141
> 3	15 (2)	12 (1)	3 (5)	**< 0.001**

Values are presented as the number of patients (%) unless indicated otherwise. Significant *p*-values (< 0.05) are highlighted in bold

### Surgical and radiosurgical management

**Fig. 2:**
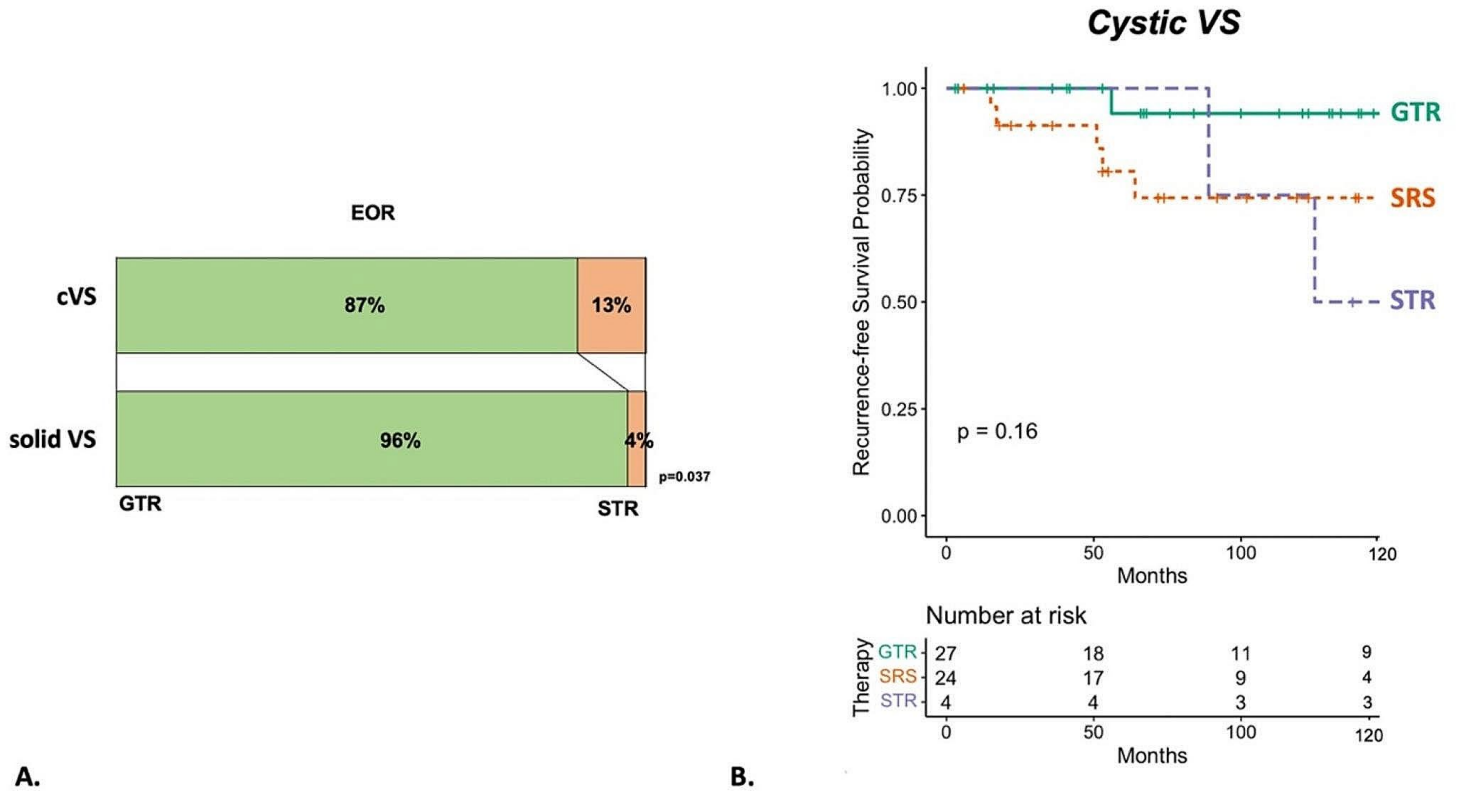
(**A**) shows the distribution in EOR in cystic VS (cVS) compared to solid VS. (**B**) shows a Kaplan-Meier-Analysis comparing time to recurrence/progression in cVS treated with GTR, STR and SRS with GTR ensuring the highest rate of tumor control

The rate of GTR was 87% in cystic VS and therefore significantly lower, compared to 96% in solid VS (*p* = 0.037) (Fig. 2). Cystic VS treated with *SURGERY* were significantly younger compared to cystic VS treated with *SRS* (*p* < 0.001). Moreover, the *SURGERY* group of cystic VS consisted of significantly larger tumor size (in Koos Classification) (Table 2). Shunt-Dependency and incidence of recurrence/progression was higher in *SRS*-treated cystic VS, but this did not reach statistical significance in this study cohort (*p* = 0.307 and *p* = 0.157 respectively). Premorbid status was statistically insignificant in cystic VS, when comparing either therapy. Mean treated tumor volume was 4.95 (± 3.28) cm^3^ with a tumor isodose volume of 4.89 (± 3.19) cm^3^.

**Table 2:** Tumor and Patient Demographics of cVS treated with *SRS* and *SURGERY*

	*SRS* (*N* = 24)	*SURGERY* (*N* = 31)	*p*-value
Age	64.33(± 14.21)	47.65(± 14.32)	**< 0.001**
Female	19 (79)	16 (52)	**0.049**
Koos Klassification			
Koos I	2 (8)	0 (0)	0.185
Koos II	11 (46)	1 (3)	**< 0.001**
Koos III	9 (38)	7 (23)	0.248
Koos IV	2 (8)	23 (74)	**< 0.001**
Shunt-Dependency	3 (13)	1 (3)	0.307
Recurrence / Progression	6 (25)	3 (10)	0.157
Charlson Comorbidities Index (CCI)	0.75(± 1.22)	0.39(± 1.17)	0.271

Values are presented as the number of patients (%) unless indicated otherwise. Significant *p*-values (< 0.05) are highlighted in bold

### Functional outcome

The overall incidence for treatment-related side-effects was 7%, with no significant difference depending on cystic morphology (*p* = 0.48). The complication rate in cystic VS treated with *SURGERY* was 6%, and 15% in solid VS (*SURGERY*) with no statistically significant difference (*p* = 0.296). The incidence of treatment-related side-effects was 0% in cystic VS treated with *SRS* and 2% in solid VS (*SRS*) (*p* = 1). The most common *SRS*-related side effects were symptomatic brain edema or hydrocephalus, while *SURGERY*-related complications were CSF fistula, hemorrhage, hydrocephalus, sinus thrombosis, symptomatic pneumocephalus, hygroma, and infection (See Table 3).

**Table 3:** Pre- and postoperative status in secondary VS-related symptoms: facial spasm, tinnitus, trigeminus, vertigo and incidence of treatment-related side effects classified in Clavin-Dindo-Classification (CDC) [21]

	All (*N* = 901)	Solid VS (*N* = 846)		Cystic VS (*N* = 55)	*p*-value
preoperative clinical status					
Facial Spasm	0 (0)		0 (0)	0 (0)	NA
Tinnitus	663 (74)		619 (73)	44 (80)	0.343
Trigeminus	94 (10)		82 (10)	12 (22)	**0.009**
Vertigo	549 (61)		504 (60)	45 (82)	**< 0.001**
postoperative clinical status					
Facial Spasm	28 (3)		28 (3)	0 (0)	0.407
Tinnitus	264 (29)		247 (29)	17 (31)	0.761
Trigeminus	54 (6)		48 (6)	6 (11)	0.133
Vertigo	359 (40)		340 (40)	19 (35)	0.478
Treatment Complications / Side Effects	62 (7)		60 (7)	2 (4)	0.577
Clavien-Dindo-Classification (CDC)					
2	21 (2)		20 (2)	1 (2)	1
3a	29 (3)		28 (3)	1 (2)	1
3b	12 (1)		12 (1)	0 (0)	1
> 4	0 (0)		0 (0)	0 (0)	NA

Values are presented as the number of patients (%) unless indicated otherwise. Significant *p*-values (< 0.05) are highlighted in bold

**Fig. 3:**
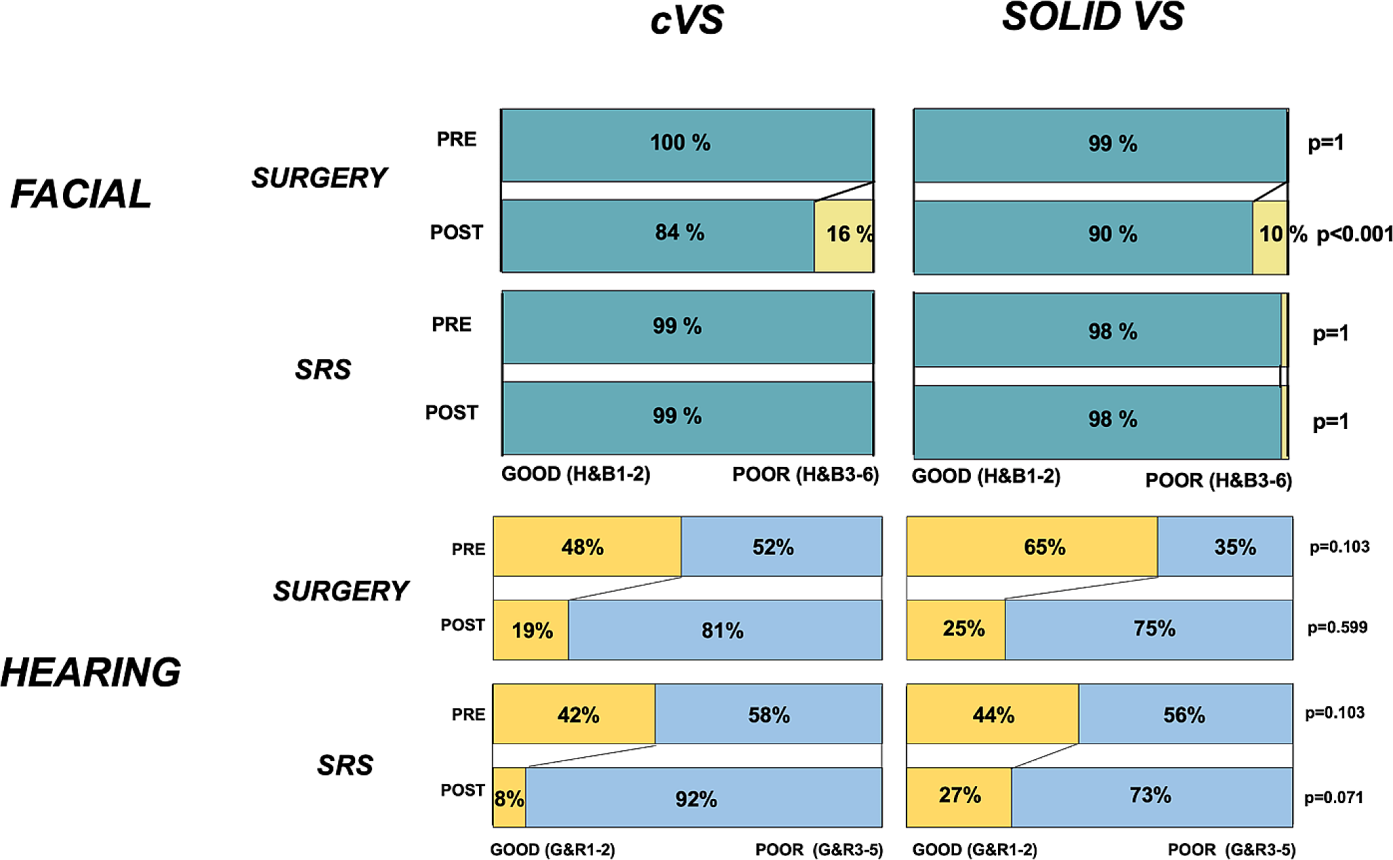
Rates of functional status in facial and hearing function in cVS (*N* = 55) and solid VS (*N* = 849) treated with either SURGERY or SRS. Good facial function was defined as H&B1-2, and poor status as H&B3-6. Good hearing status was defined as G&R1-2, and poor status as G&R3-5. Values are presented as the number of patients (%) unless indicated otherwise. Significant *p*-values (< 0.05) are highlighted in bold

Post-operative facial and hearing function was similar in cystic VS compared to solid VS. Postinterventional facial function was significantly worse in patients treated with *SURGERY* compared to *SRS* (Fig. 3). Still, within the *SURGERY*-treated VS, patients with cystic VS more often presented with relevant post-operative facial function.

### Matched comparison subgroup analysis

To confirm/deny the hypothesis that cystic fared worse after *SURGERY* compared to their solid counterparts, we conducted a subgroup-analysis with a matched cohort (1:1) of solid VS (*N* = 31) with similar tumor characteristics as cVS treated by *SURGERY*- as the tumor size distribution were statistically different in the cystic and solid tumors. Here, the incidence of relevant, post-operative long-term facial palsy was 23% in a comparative solid VS group (compared to 16% in cVS treated by *SURGERY*) (*p* = 0.749). Shunt-Dependency was similar in the matched solid VS groups with 6% compared to 3% cVS (*p* = 0.350). Same applied to the incidence of pre- and post-operative vertigo and hearing function: Neither was significantly higher in cystic VS compared to solid VS, when the factor of tumor sizing was dissolved (74%; *p* = 0.780 and 58%; *p* = 0.611).

A direct matched comparative subgroup analysis was also performed in the *SRS*-group. The rate of long-term tumor progression was higher in cVS (25%) treated by *SRS* compared to same-sized sVS (13%), however, it did not reach statistical significance (*p* = 0.461). The pattern of volume change within the first 2 years after *SRS*-treatment in this subgroup-analysis is shown in Table 4. The incidence of dynamic volume change (tumor volume decrease, pseudoprogression, or volume increase was significantly more common in cystic VS compared to the matched solid VS (*p* = 0.042).

**Table 4:** Incidence of different patterns of volume change within 2 years after SRS in cystic VS compared to matched solid VS

	Cystic VS (*N* = 24)	matched solid VS (*N* = 24)	*p*-value
same tumor size (± 30%)	8 (33)	16 (66)	0.042*
tumor volume decrease (> 30%)	7 (29)	3 (13)	0.286
pseudoprogression	6 (25)	1 (4)	0.097
early tumor progression	3 (13)	4 (17)	1

Values are presented as the number of patients (%) unless indicated otherwise. Significant *p*-values (< 0.05) are highlighted in bold

### Tumor control

Overall mean time of FU was 6.69 (± 4.44) years in cystic VS and 6.23 (± 4.36) years in the overall study cohort. *SURGERY* as a monotherapy was able to ensure a comparable tumor control in cystic VS compared to solid VS. However, *SRS* showed inferior tumor control in cystic VS with 25% treatment failure compared to *SRS*-treated solid VS with 10% treatment failure (*p* = 0.014). Incidence rates are shown in Table 5. When comparing EOR in the *SURGERY-*treated cystic VS, the rate for tumor recurrence/progression was significantly lower in GTR with 4% compared to STR with 50%, and *SRS* with 25% (*p* = 0.042; *p* = 0.037), making GTR the best treatment choice considering tumor control (compared to *STR* and *SRS*).

**Fig. 4:**
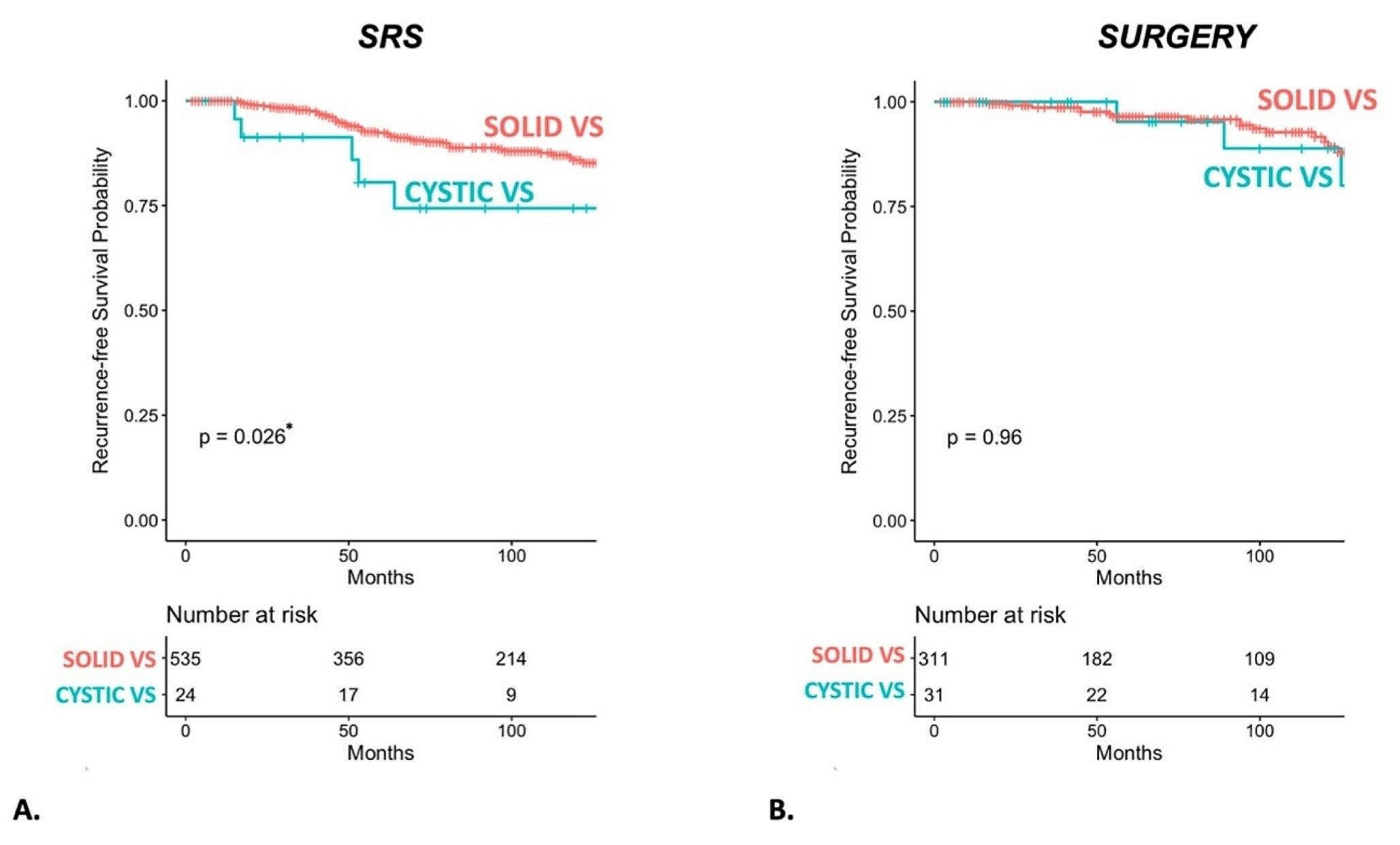
(**A**) shows a Kaplan-Meier-Analysis comparing progression-free-survival in cVS and solid VS, when treated with *SRS*. (**B**) shows recurrence-free-survival in cVS and solid VS when treated with SURGERY.

In GTR-treated cystic VS (*N* = 1), time to recurrence was 4.67 years. When treated with STR, mean time to recurrence was 8.92 (± 2.12) years. In *SRS-*treated cystic VS, mean time to recurrence was significantly shorter compared to STR at 5.04 (± 4.51) years (*p* < 0.001). Mean time to tumor progression of *SRS*-treated cystic VS was statistically insignificant compared to solid VS treated with *SRS* 6.37 (± 4.33) years (*p* = 0.142). Kaplan-Meier-Analysis on *SRS* and *SURGERY* in solid versus cystic VS is shown in Fig. 4.

**Table 5:** Incidence of recurrence/progression in solid VS and cVS treated with SURGERY or SRS

	Solid VS (*N* = 846)	Cystic VS (*N* = 55)	*p*-value
SRS	10% (*N* = 53/535)	25% (*N* = 6/24)	**0.014**
SURGERY	7% (*N* = 22/311)	10% (*N* = 3/31)	0.484
*p*-value	0.170	0.157	

Values are presented as the number of patients (%) unless indicated otherwise. Significant *p*-values (< 0.05) are highlighted in bold

## Discussion

From all treated solitary, primary VS, 6% showed MR-graphic cystic character in this study. The majority of cystic VS was treated with *SURGERY*. Premorbid status was worse in cystic compared to solid VS. Generally, cystic VS were larger compared to solid VS and more often required hydrocephalus-related treatment (i.e. shunt surgery). The incidence of pre-operative trigeminal and vertigo-related symptoms are significantly more frequent in cystic VS compared to solid tumors – but not higher compared to same-size solid VS. *SRS* was inferior in tumor control (i.e. RFS) in cystic VS compared to solid VS. The highest rate of tumor control was ensured, when treated with GTR (compared to *SRS* and STR). The rate of GTR, however, is significantly lower in cystic VS compared to surgically treated solid VS. In the general cohort, poor postoperative facial outcome was significantly more prevalent in cystic VS, but not worse compared to same-size solid VS. Cystic morphology was not associated with a higher rate of therapy-related side-effects.

### Patient and tumor characteristics solid vs. cystic VS

The incidence of cystic morphology in the VS is reported with considerable variability: from 11.3 to 48% [9, 22, 23]. This cohort shows an overall incidence of 6% of the treated primary, solitary VS similar to Fundova et al. in 2000, when 773 VS patients were retrospectively reviewed [24]. However, the incidence of cyst formation rises with tumor size with the lowest rate in Koos I at 2% and the highest incidence in Koos IV VS at 15.43%, this distribution could attribute to the reported incidence discrepancies [25]. 

The CCI was significantly higher in patients with cystic VS compared to solid, suggesting that there was a significantly worse premorbid status in these patients, even though age is indifferent in either group. Even though the exact pathophysiology of cyst formation is not entirely clear, it has been related to micro-hemorrhages and inflammation process and one could imagine, this being a possible effect of the patients’ overall health condition or relevant comorbidities [8, 26]. 

The incidence of trigeminal symptoms and vertigo was significantly raised in cystic VS compared to solid VS. This has also been shown and discussed by Constanzo et al. in 2019, who showed that patients with cystic VS faired objectively worse in video head impulse testing and therefore was associated with worse vestibular dysfunction [26]. Constanzo et al. attribute this difference to a local inflammatory reaction caused by hemorrhages, which would induce a ‘‘neuritis’’ of the vestibular nerves, therefore altering vestibulo-ocular reflex gain beyond what its expected [26]. However, in our matched subgroup-analysis, where tumor size was taken out of the equation as a potential bias, there was no significant difference in vertigo, trigeminal affection, hearing status and even facial nerve outcome. This suggests that the pronounced clinical features of a cystic VS is most likely caused by the sheer space demanding tumor volume in the CPA. The incidence of Shunt-Dependency was also significantly higher in the cystic VS cohort, which has been described in the past [25, 27]. 

### Radiosurgery vs. microsurgery

Patients treated by *SRS* were significantly older compared to cystic VS treated with *SURGERY*, which is a phenomenon often described in the past [14, 28–30]. Interestingly, women more often received *SRS* than men. However, this study design does not allow any investigation to any sex-related difference in provided VS care. However, differences in provided surgical care have been described in the past – although it remains unclear, whether this phenomenon is a result of medical care provider bias or gender-related decision-making by the patient [31, 32]. 

It has been repeatedly shown in the past that *SRS* is safe in cystic VS with a high rate of functional preservation [1, 4, 12, 33]. Within the *SRS*-group, however, it was not able to ensure the same RFS in cystic VS as in solid VS (75% versus 90%). When treated with *SRS*, the incidence of post-*SRS* tumor volume change (increase, decrease or pseudoprogression) was significantly higher in cystic VS in the matched subgroup analysis. This phenomenon has been described by previous studies focusing on *SRS* in cystic VS, who describe a significant tumor volume decrease in macrocystic compared to solid VS [1, 12, 34, 35]. 

Bowden et al. observed a very high tumor control rate of > 95% (compared to 75% in this study) with a mean follow-up of 4 years. However, our data shows a mean time to recurrence/progression in the *SRS*-treated cystic VS at > 5 years. It is possible that past studies have not been able to report the true incidence of recurrence, when cystic was treated with *SRS* as a monotherapy [4, 12, 13]. In 2016, Frisch et al. reported a tumor control of 80% in cystic VS with a mean follow-up of 5.25 years, which more reflects to the results presented in our study [1]. These differences point out the importance of long-term FU when discussing RFS in a benign tumor, such as VS.

To our knowledge, this is the first study to directly compare *SRS* and *SURGERY* of cystic VS in one study design. Noticeably, if tumor recurrence/progression appeared, mean time to recurrence/progression after *SRS*-treatment was significantly shorter compared to STR or GTR – even though pseudoprogression was systematically ruled out in this study. Therefore, the tumor volume increase in cystic VS is most likely due to the fluid uptake in the intratumoral cyst and not due to increase in contrast-enhancing tumor tissue. Concordant, cystic schwannomas are histologically associated with a 36-fold decrease in nuclear proliferation as measured with Ki-67 staining when compared with solid tumors. This suggests that the rapid clinical growth seen in cystic schwannomas is related to the accumulation of fluid during cyst formation and not by an actual increase in the growth rate of tumor cells [3, 36]. 

### The predicament of surgery of cystic VS

The prognosis after *SURGERY* is reported to be worse than for other solid VS because of the difficulty in preserving the arachnoid plane, the presence of hypervascular solid portions of the tumor, unusual cranial nerve displacement, and a greater tendency for postoperative bleeding [5, 6, 8, 37, 38]. Therefore, cystic VS presents a therapeutic dilemma and should preferably be treated in specialized centers routinely treating solid VS.

Safe maximal resection—if achievable—would to date be the best long-term tumor control in cystic VS according to our results. However, cystic morphology is associated with a lower rate of EOR (GTR 87% vs. 96%), when treated with *SURGERY* in this study cohort. A comparative study in 2005 achieved a high GTR-rate in cystic tumors of 92% (solid tumors 93%) by retrosigmoid approach, but they also report that 42% of patients with cystic VS showed unfavorable facial nerve function one year post-operatively [9]. Notably, their classification for good facial outcome included HB grade 3, which was classified as poor facial outcome in our study. In 2000, Fundova et al. reported a substantially higher rate of complete facial nerve loss in cystic compared to solid VS (41% versus 27% respectively) with similar GTR rates in both groups (89% and 82%) [24]. 

The rate of poor facial outcome (HB3-6 after one year) was rather low with 16% in cystic VS and comparable in same sized solid VS in our study compared to the literature. Jian et al. presented a cohort with significantly different achieved EOR with 52% of GTR in cystic and 70% in solid VS, this constellation yielded in insignificantly different facial function outcome in either group [39]. In summary, studies on cystic VS yielded in comparable facial nerve outcome, when the rate of STR was higher compared to solid VS suggesting a direct correlation between EOR and facial nerve outcome [7, 11, 39–41]. 

A trend towards a more conservative surgical approach, which has also been reported in a longitudinal analysis by Piccirillo et al. in 2009, may conceal the worse facial function resulted by the cystic morphology [42]. Facial function has been shown to be a major predictor of VS patients’ quality of life [28]. Along with the unfavorable prognosis after *SRS*, it is enticing to conclude that a combination approach, e.g. STR with cyst resection followed by STR in cystic VS might ensure best facial nerve outcome and tumor control [2, 43]. Still, before putting these kinds of recommendations forward, further investigation must be done, on how the solid residual tumor reacts to *SRS.* It has been described that *SRS* may cause the micro-hemorrhages and therefore, could promote new cyst formation in tumor tissue already prone to cyst formation [6, 44, 45]. Yang et al. have shown retrospectively that adjuvant *SRS* after complete cyst removal ensures higher tumor control rates then in solid VS, however, their mean time of follow up was 4.5 years, which for a benign tumor such as VS may be too short of a follow-up [43]. A prospective study on a combination approach in cystic VS has yet to be done. As STR significantly prolongs mean time to recurrence to 8.92 years, STR can be a valid option for Elderly patients with cystic VS.

### Limitations of this study

This study is limited by its nature of retrospective design, even though it involves more than one study center. This study involved a large group of patients with cystic tumor characteristics, however, the patient number and its value has to be put into its statistical context. An even larger patient cohort could even allow subgroup analyses within cystic VS (e.g. micro- and macrocystic morphology).

## Conclusion

Tumor control in cystic VS is superior with *SURGERY*, when treated with a high EOR, compared to *SRS*. However, when cystic VS is treated surgically, the rate of GTR is lower and the number of patients with relevant postoperative facial palsy higher compared to solid VS in general, but not higher compared to same-sized solid VS. Therefore, cystic morphology in VS poses a challenge in the management (both *SRS* and *SURGERY*) compared to solid tumors. Treatment decision and patients’ consultation should contemplate and address negative prognostic factor of cystic morphology in VS.

## Data Availability

No datasets were generated or analysed during the current study.

## Abbreviations

ARI: Absolute Risk Reduction
CCI: Charlson Comorbidities Index
CDC: Clavien-Dindo-Classification
CTC: Clinical tumor control
DS: Decompression surgery
ENT: Ear, Nose and Throat
EOR: Extent of resection
FU: Follow-up
GKR: Gamma-Knife radiosurgery
GR: Gardner-Robertson
GTR: Gross total resection
H&B: House-Brackman
KPS: Karnofsky Performance Score
MRI: Magnetic resonance imaging
N/A: Not applicable
RTC: Radiographic tumor control
SRS: Stereotactic radiosurgery
ST: Salvage therapy
STR: Sub-total resection
TV: Tumor volume
VS: Vestibular schwannoma
